# Force of Medical Delusion

**Published:** 1846-06-01

**Authors:** 


*Force of Medical Delusion.*—The following anecdote was related by M. Majendie, in opening a course of lectures on Physiology at the College of France; we quote from an extract in the Boston Medical and Surgical Journal:

"A lady, a fervent believer in mesmerism, asked her niece, who was poorly, for a lock of her hair, in order to consult a somnambulist. The niece, wishing to try the credulity of her aunt, gave her the hair of her maid instead of her own. A renowned somnambulist was consulted, and at once recognized, by the hair of the maid, all the symptoms presented by the niece, whose sufferings she minutely described, to the great edification of the lady. The latter was then informed of the trick that had been played. You would naturally have thought that she would have recognized the imposture of the somnambulist. Not at all; she preferred concluding that the maid servant had the same disease as her niece, and obliged her to submit to a regular treatment, as if she had been poorly, although at that time in the best possible health."

